# The CHESS trial: protocol for the process evaluation of a randomised trial of an education and self-management intervention for people with chronic headache

**DOI:** 10.1186/s13063-019-3372-x

**Published:** 2019-06-04

**Authors:** Vivien P. Nichols, David R. Ellard, Frances E. Griffiths, Martin Underwood, Stephanie J. C. Taylor, Shilpa Patel, and on behalf of the CHESS team, Arlene Wilkie, Brendan Davies, David Boss, David Ellard, Dawn Carnes, Felix Achana, Fiona Caldwell, Frances Griffiths, Harbinder Sandhu, Helen Higgins, Hema Mistry, Katrin Probyn, Kimberley White, Kirstie Haywood, Manjit Matharu, Martin Underwood, Mary Bright, Rachel Potter, Sandra Eldridge, Shilpa Patel, Siew Wan Hee, Simon Evans, Stavros Petrou, Stephanie Taylor, Tamar Pincus, Vivien Nichols

**Affiliations:** 1 Warwick Clinical Trials Unit, Division of Health Sciences, Warwick Medical School, University of Warwick, Coventry, CV4 7AL UK; 2 Division of Health Sciences, Warwick Medical School, University of Warwick, Coventry, CV4 7AL UK; 3 Complex Intervention and Social Practice in Health Care unit, Centre for Primary Care and Public Health, Blizard Institute, Barts and The London School of Medicine and Dentistry, Queen Mary University of London, London, E1 2AB UK

**Keywords:** Process evaluation, Chronic headache, Self-management, Chronic migraine, Chronic tension type headache and medication overuse headache

## Abstract

**Background:**

Process evaluation is increasingly common alongside complex randomised controlled trials (RCTs). This evaluation helps in understanding the mechanisms of impact and how the study processes were executed, and it includes any contextual factors which may have implications for the trial results and any future implementation. This process evaluation is for the Chronic Headache Education and Self-management Study (CHESS) RCT, which is evaluating an education and self-management group behavioural intervention for people with chronic headache. Chronic headache is defined as headaches which are present for 15 or more days per month. The most common types are chronic migraine and chronic tension type and medication overuse headaches.

**Methods:**

We will use a mixed methods approach. Quantitative data will be taken from routine trial data which will help us to assess the reach of the study; i.e. did we reach those whom we expected and from where? Intervention attendance (dose received) and attrition and qualitative data will augment our understanding about reasons why people may not wish to take part in or failed to attend sessions. Interviews with intervention facilitators and trial participants will gain different perspectives on taking part in the trial.

Fidelity will be assessed through listening to audio recordings for adherence to course content and competence of the facilitation of a sample of sessions.

**Discussion:**

Our process evaluation will allow us to gain insight into how the trial was delivered, the obstacles and enablers encountered and the possible reasons why the interventions may or may not be effective.

**Trial registration:**

ISRCTN79708100. Registered on 16 December 2015.

**Electronic supplementary material:**

The online version of this article (10.1186/s13063-019-3372-x) contains supplementary material, which is available to authorized users.

## Background

Health research interventions are becoming increasingly complex, encompassing many interconnecting and interacting components [1]. The primary outcome is the core focus of randomised controlled trials (RCTs) of complex interventions in health care. Nevertheless, consideration of the study processes, including design and execution, can be equally important. Evaluation of the principal processes of a complex study assists in elucidating why a particular intervention is, or is not, successful in achieving its outcome. It may also help to explain how an intervention could be optimised or why it may have failed [1–3]. There is now a growing body of published process evaluations that help to put trial results into context [4–8]. Having a clear understanding of the processes of a study is also very helpful for study replication and for informing future research.

Key components of process evaluation, including reach, recruitment, treatment fidelity, dose delivered and dose received, help to define the extent to which an intervention is implemented [3]. This structured process helps to identify problems and factors that may have caused a deviation in expected outcomes. For example, it may highlight contextual factors which could have impacted the trial implementation, which might not otherwise have become apparent.

The Chronic Headache Education and Self-management Study (CHESS) has a full study protocol compliant with Standard Protocol Items: Recommendations for Interventional Trials (SPIRIT) guidelines, which is available including checklist and figure. Within this protocol is an outline of the process evaluation planned for CHESS (Patel S, et al., Usual Care and a Self-Management Support Programme vs Usual Care and a Relaxation Programme for People Living with Chronic Headache Disorders: A Randomised Controlled Trial Protocol (CHESS), submitted). To ensure scientific rigour (in research conduct and reporting) we present here a more detailed protocol for the process evaluation being undertaken within CHESS. This protocol, as a component of the main CHESS protocol, does not fit within the SPIRIT framework, as it is not a full interventional trial protocol.

In the CHESS study chronic headache is defined as one which is present for 15 or more days per month for at least 3 months [9]. It affects between 3 and 4% of the population [10]. Medication overuse headache (medication overuse being both a consequence of, and a cause of, chronic headache) affects between a quarter and a half of those with chronic headaches. Despite the scale of the disability associated with chronic headache worldwide [11], there have been very few studies exploring how to support people to manage their headaches better. The CHESS trial is testing the impact of a supportive group self-management programme on the headache-related quality of life in people living with chronic headaches.

Before entering the CHESS trial all participants take part in a headache classification interview via telephone (Potter R, et al., Development and Validation of a Telephone Classification Interview for Common Chronic Headache Disorders, submitted). This ensures all participants have chronic migraine (CM) or chronic tension type headache (CTTH), in the presence or absence of medication overuse headache (MOH). All study participants and their general practitioners (GPs) are given current evidence-based advice about their type of headache and medication options. The CHESS intervention arm consists of a 2-day educational and self-management group intervention held in a local community setting, followed by an individual face-to-face session. In this session a nurse discusses the participant’s classification, medication options and the personal goals they have or plan to set. They will then mutually agree on the amount of telephone support they would like, over the following 8 weeks. The control arm patients receive usual care plus a relaxation CD. They and their GPs also receive feedback on the classification interview. More detailed information on the CHESS study can be found in the previously mentioned study protocol of S. Patel et al.

The trial and the process evaluation described are both funded by a National Institute for Health Research (NIHR) programme grant, project number RP-PG-1212-20018 (https://www.journalslibrary.nihr.ac.uk/programmes/pgfar/RP-PG-1212-20018/#/). Ethics approval has been granted for the trial and its process evaluation by the North West-Greater Manchester East Research Ethics Committee*.* The trial registration number is ISRCTN 79708100.

### Formative process evaluation pre main trial

A formative process evaluation undertaken during the feasibility study preceding the main trial was used to refine intervention delivery and recruitment procedures in the main trial. Data collection included recruitment rates and observations and interviews with study participants and facilitators. Participants enjoyed meeting other people with chronic headaches in a group setting, but at interview suggested reducing the length of the group intervention due to the pressures of work and family commitments. This was also a major reason given for being unable to take part in the feasibility study and/or attending the group intervention. Following participants’ suggestions, the course duration and content were reduced to 2 days separated by a week instead of having 2 consecutive days followed by a shorter day a week later [12]. The group session content was well received, and even when elements were not personally useful, interviewees felt that all the content was potentially useful to others. Some talked of gaining new knowledge, and others had tried some of the techniques suggested. The trial paperwork (e.g. questionnaires and diaries) was not considered to be a burden.

The intervention group facilitators felt they needed further training and guidance on medication for headaches. This training was expanded for the main study. Lay facilitators with experience of frequent headaches helped to deliver the feasibility group intervention. However, they were always uncertain as to whether they would be able to attend on a scheduled day due to the unpredictability of their headaches. The team reluctantly made a pragmatic decision to use nurses with other allied health professionals only to deliver the intervention in the main study. In the following subsections we present the aims and objectives of the main CHESS process evaluation.

### Aims

Here we present the protocol for the process evaluation of the CHESS RCT.

The aims of the process evaluation are:

1. To assist in the interpretation of the results of the main effectiveness trial
2. To develop a set of transferable principles regarding the intervention to inform its implementation on a wider scale, if the intervention proves effective.

### Objectives

Our specific objectives are:

- To monitor implementation processes, i.e. recruitment, reach, dose delivered, dose received, delivery of the intervention and acceptability/use of the intervention in practice and fidelity
- To explore participants’ experiences of living with chronic headaches whilst taking part in the trial and to explore any ongoing use and experience of the intervention through in-depth interviews
- To explore with members of the recruitment team and the intervention delivery teams their experiences and possible facilitators and barriers to wider implementation.

## Methods

### Rationale for our approach

**Fig. 1:**
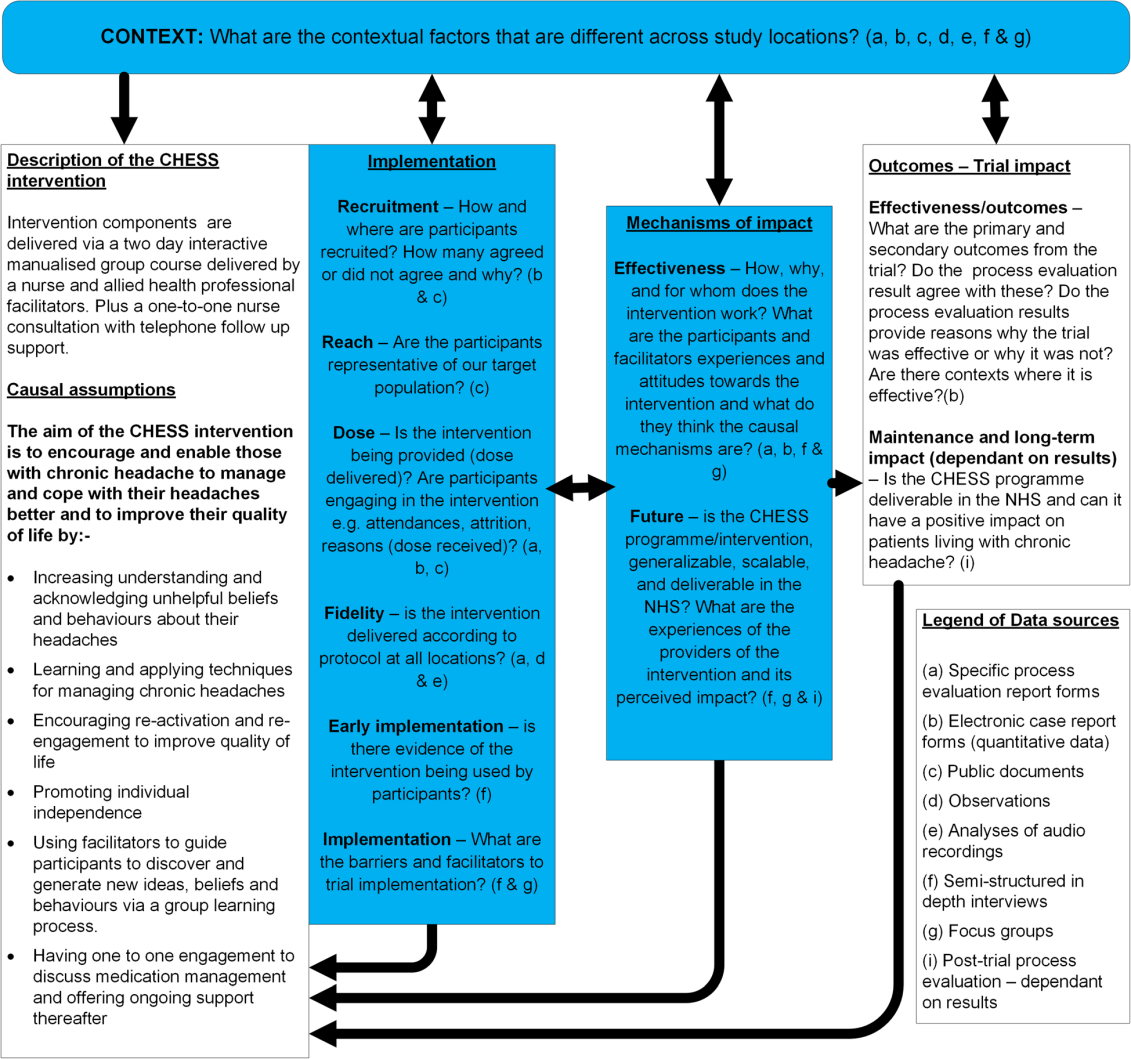
The CHESS process evaluation. The process evaluation components are highlighted in the *shaded boxes*. Informed by the intervention description which informs interpretation of outcome (adapted from Moore et al., [1])

The process evaluation has been developed within a framework based on the Medical Research Council (MRC) guidance and incorporates components of the process evaluation model proposed by Steckler and Linnan, which includes context, reach, dose delivered, dose received, fidelity and recruitment [1, 3]. We are adopting a mixed methods approach for the process evaluation [13–15]. The principal data collection method will be quantitative, whilst qualitative data will complement the quantitative data, providing a depth and breadth of understanding. We will add to this an exploration of the experience of delivering and receiving the intervention to inform any future rollout of the intervention and the early impact of the intervention. In Fig. 1 we present the key functions of the CHESS process evaluation. Whilst it appears to be a linear progression, feedback loops between components within the framework may occur at all stages, as illustrated by the black arrows. This reflects the process where intervention descriptions and causal assumptions may need revisiting. Emerging insights into mechanisms triggered by the intervention may have an impact on implementation.

The process evaluation within the CHESS study has a dedicated team who are part of the overall team but who are not involved in delivering the intervention or measuring its effectiveness. The team will meet regularly and will be responsible for the process evaluation, data collection, analysis and final report.

The data from this summative process evaluation will be analysed separately from the main trial results and presented before the main outcomes are reported. It will contribute to a review of the a priori analysis plan before trial analysis commences [13].

### Quantitative data

To determine the study recruitment reach and context, we will collect routine trial recruitment data to assess if we reached our populations of interest across different geographic locations. We will also collect data, where possible, on why people do not want to participate in the study from the expression of interest forms (which will include a number of options participants can choose if they do not want to participate) or why people who initially want to take part may subsequently be unable to participate (e.g. from notes to file following contact with a participant who drops out).

### Qualitative data

A focus group or individual interviews will be carried out with recruitment staff to ascertain facilitators and barriers to recruitment.

### Intervention delivery

#### Quantitative data

To determine the dose delivered and the dose received, we will collect data on the numbers and locations of the groups run. We will use attendance sheets to collect which group sessions and individual sessions were attended and how many of the support telephone calls were received. Table 1 lists the quantitative data sources.

**Table 1:** Quantitative data sources

Item	Source	Description
Dose delivered	Trial intervention data	Numbers of groups delivered/not delivered and why, location of groups
Dose received	Trial intervention attendance sheets for groups, one-to-one nurse consultations and telephone calls	Attendance and attrition data

#### Qualitative data

We will use semi-structured interviews with participants and focus groups or interviews with intervention staff to ascertain their experiences of being in the study. The qualitative data will be used to enhance the quantitative data and provide insight into how the intervention may or may not have been accessed, utilised and/or interacted with. Our decision to offer staff the option of interview or focus group was a pragmatic and practical one. Our preference is the group setting, as it will give the opportunity for discussion from different perspectives as participants reflect on their experiences. However, gathering groups of people together has its problems, so those who cannot attend will be offered an interview so that they too can have a voice.

#### Participant interviews

All consenting participants in the main trial will indicate if they would be willing to be approached for an interview. The process evaluation team will select and invite a purposive sample of these participants based on their headache type.

To understand the participant’s journey, we will carry out semi-structured interviews with a purposive sample of approximately 30 participants from both the usual care and intervention arms at three time points. Trial participants will be sent a separate patient information leaflet explaining the purpose of the interviews. They will then be contacted a week later, and any questions will be answered. Consent will be taken at the first interview but reconfirmed verbally before each subsequent interview. Interviews will be performed face to face at the first two interviews and by telephone at the last. The first interview will be held after consent and classification but prior to randomisation, the second and third after participants have returned their 4 and 12 months questionnaires respectively. We will use framework analysis to interpret the data through a phenomenological lens seeking to understand the participants’ experience as they move through the different stages of the research [15, 16]. First we will study their present lived experience of headaches and their expectations of being on the study, then their experiences of engaging (or not) with the study and finally their view of any long-term impact. Issues or comments raised at the first interview will be addressed at subsequent interviews, and data analysis will take place iteratively throughout data collection.

All intervention staff will be invited to be interviewed individually or in focus groups at the end of the intervention delivery. We will also ask participating GP practices to complete a feedback form. Table 2 outlines the qualitative data, its purpose and its time points within the study.

**Table 2:** Qualitative data purposes and time points

Stakeholder	Purpose	Time point
Participant’s 1st	To explore:	Post consent and pre
interview	• Expectations of the CHESS intervention	randomisation
	• Experiences of living with chronic headaches	
	• Trial experiences	
Participant’s	To explore:	Post 4 months questionnaire
2nd interview	• Reflection on trial experiences	
	• Current headache experiences	
	• Use of the trial materials	
	• Early impact	
Participant’s	To explore:	Post 12 months questionnaire
3rd interview	• Further reflection on trial experiences	
	• Current headache experiences	
	• Use of the trial materials	
	• Early impact	
Intervention staff	To understand their experiences of delivering the intervention	End of all trial intervention delivery
GP feedback	To understand their trial experiences	End of practice patient’s involvement

Qualitative work will add to the richness of the data by providing a deeper understanding of what the participants thought about attending the course, including whether they learned anything new or changed their management of their headaches specifically by changing their medication and/or behaviour.

### Fidelity

Fidelity will be assessed from audio recordings of a selection of group sessions. Key sessions from the whole course were identified by the intervention development team in collaboration with the process evaluation team. These sessions were considered to be the most likely to promote behaviour change and deliver new knowledge (see Table 3). The other sessions concerned practical techniques and lifestyle advice. An overall impression of the course as a whole will be explored through participant and staff interviews and feedback.

**Table 3:** Key components of the group intervention

Day	Session number	Session name
1	3	Headache information and mechanisms
1	4	Acceptance of chronic headaches
1	5	Impact of thoughts, mood and emotions on headaches
1	6	Headache cycle and breaking the cycle
1	7	Unhelpful thinking patterns and finding alternatives
2	10	Identifying barriers to change and exploring problem solving and goal setting
2	17	Communicating better with health care professionals
2	18	Managing setbacks: what to do when things don’t go according to plan

**Table 4:** The CHESS team

Name	Role	Organisation
Professor Martin Underwood	Chief Investigator	Warwick Clinical Trials Unit
Ms Mary Bright	Co-applicant (National Health Service Manager)	University Hospitals Coventry and Warwickshire
Dr Dawn Carnes	Co-applicant	Queen Mary University London
Dr Brendan Davies	Co-applicant	Royal Stoke University Hospital
Professor Sandra Eldridge	Co-applicant	Queen Mary University London
Dr David Ellard	Co-applicant	Warwick Clinical Trials Unit
Simon Evans	Co-applicant	Migraine Action
Professor Frances Griffiths	Co-applicant	Warwick University, Division of Health Sciences
Dr Kirstie Haywood	Co-applicant	Warwick University, Division of Health Sciences
Dr Siew Wan Hee	Co-applicant	Warwick University, Division of Health Sciences
Dr Manjit Matharu	Co-applicant	National Hospital for Neurology and Neurosurgery
Professor Stavros Petrou	Co-applicant	Warwick University, Division of Health Sciences
Professor Tamar Pincus	Co-applicant	Royal Holloway
Dr Harbinder Sandhu	Co-applicant	Warwick Clinical Trials Unit
Professor Stephanie Taylor	Co-applicant	Queen Mary University London
Arlene Wilkie	Co-applicant	The Migraine Trust
Helen Higgins	Senior Project Manager	Warwick Clinical Trials Unit
Vivien Nichols	Researcher	Warwick Clinical Trials Unit
Dr Shilpa Patel	Researcher	Warwick Clinical Trials Unit
Dr Rachel Potter	Researcher	Warwick Clinical Trials Unit
David Boss	Data Entry Clerk	Warwick Clinical Trials Unit
Kimberley White	Trial Coordinator	Warwick Clinical Trials Unit
Felix Achana	Researcher	Warwick University, Division of Health Sciences
Hema Mistry	Researcher	Warwick University, Division of Health Sciences
Fiona Caldwell	Researcher	Queen Mary University London
Katrin Probyn	Researcher	Royal Holloway

Using a random number generator in Microsoft Excel (2007), we created two randomisation tables (allowing for up to 40 groups in the Midland area and 20 groups in the London area to be delivered). To ensure all groups were represented and to minimise bias, groups were numbered consecutively, and three sessions and a back-up session were randomly assigned to each group.

One member of the process evaluation team will listen to the audio recorded sessions and will rate them on adherence (whether they delivered the content as per the manual) and competence (how well the course leaders facilitated the sessions). The marking criteria will be evident, partially evident and not evident. Scores of 2/1/0 respectively will be given. A second researcher will rate 10% of these sessions to ensure inter-rater reliability and credibility of the scoring process.An example of an assessment table is given in Additional file 1.

Initially we were going to observe 10% of the intervention groups and collect data specifically about the group dynamics and the sessions not covered in the fidelity assessments to gain an overall impression of the intervention. We decided to obtain this information from the staff and group participants at interview to gain more information across a potentially larger number of group interventions.

One-to-one nurse consultations follow a set format with a structured form. On listening to two sessions at three time points (early, middle and late in the trial), we decided that the forms were comprehensive in collecting information about whether the key components of headache classification, medication and goal setting were discussed. We will look at a random sample of 10% of these forms to assess adherence and note the uptake of follow-up telephone calls numerically as well qualitatively in any comments given at interview.

### Early impact

Questions within the second and third interviews with participants will explore to what extent the participants are making use of the intervention materials now that they are approaching the end of the study. The interviewers will ask the participants for any real examples of what, when and how they are using the materials now and their experiences of whether these materials may or may not have been integrated into their lives (e.g. whether there is an impact on their headaches and or quality of life).

### Pre RCT results

It is the aim of the process evaluation team to provide a process evaluation report before the main trial effectiveness results are revealed. This will be presented to the Chief Investigator and the Trial Management Group for consideration. Dependent on the outcomes, there may be recommendations for possible subgroup analyses.

### Data analysis

Quantitative data will be entered onto the study database, and appropriate descriptive statistics, charts, tables or figures will be produced.

Qualitative data including interviews and feedback forms will be analysed using the framework method proposed by Ritchie and Spencer [16]. We will use framework analysis and comparative analysis to interpret the data as participants move through the different stages of the research. The interviews will be audio recorded on an encrypted digital recorder and transcribed verbatim; recordings will be anonymised before analysis. All data will be kept in a digitally safe environment.

## Discussion

Over the last few years the importance of a well-conducted process evaluation of complex trials has increased. Trials now go much further than determining effectiveness of an intervention. There is now a need to know and understand how or why an intervention was successful or not. Process evaluations have to be a fundamental consideration during trial development, and to ensure high standards of research conduct and reporting, clear protocols should be produced and made available in the public domain during the trial development. Here we have produced a more detailed process evaluation protocol taken from the CHESS main study protocol to provide a deeper understanding of exactly what we are planning to do and how we will report it. This we hope will help us to interpret the trial results and place them in context.

In summary, this process evaluation will explore the trial processes of CHESS and show whether the trial was delivered as intended and whether there were factors which affected its implementation.

### Trial status

The main CHESS trial started recruitment in mid-2017, and recruitment concluded at the end of March 2019.

## Additional files

Additional file 1: Process evaluation protocol for the CHESS trial: supplementary material. (DOCX 19 kb)

## Abbreviations

AHP: Allied health professional
CHESS: Chronic Headache Education and Self-management Study
GP: General practitioner
MRC: Medical Research Council
NICE: National Institute for Health and Care Excellence
RCT: Randomised controlled trial

## References

[1] Moore GF, Audrey S, Barker M, Bond L, Bonell C, Hardeman W, Moore L, O'Cathain A, Tinati T, Wight D (2015). Process evaluation of complex interventions: Medical Research Council guidance. BMJ.

[2] Baranowski T, Stables G (2000). Process evaluations of the 5-a-day projects. Health Educ Behav.

[3] Steckler A, Linnan L (2002). Process evaluation for public health interventions and research.

[4] Demment MM, Graham ML, Olson CM (2014). How an online intervention to prevent excessive gestational weight gain is used and by whom: a randomized controlled process evaluation. J Med Internet Res.

[5] Ellard DR, Taylor SJ, Parsons S, Thorogood M (2011). The OPERA trial: a protocol for the process evaluation of a randomised trial of an exercise intervention for older people in residential and nursing accommodation. Trials.

[6] Gotlib Conn L, McKenzie M, Pearsall EA, McLeod RS (2015). Successful implementation of an enhanced recovery after surgery programme for elective colorectal surgery: a process evaluation of champions’ experiences. Implement Sci.

[7] Oakley A, Strange V, Stephenson J, Forrest S, Monteiro H (2004). Evaluating processes: a case study of a randomized controlled trial of sex education. Evaluation.

[8] Partridge SR, Allman-Farinelli M, McGeechan K, Balestracci K, Wong ATY, Hebden L, Harris MF, Bauman A, Phongsavan P (2016). Process evaluation of TXT2BFiT: a multi-component mHealth randomised controlled trial to prevent weight gain in young adults. Int J Behav Nutr Phys Activ.

[9] The International Classification of Headache Disorders. https://www.ichd-3.org/. Accessed 19 Nov 2018.

[10] Stovner L, Hagen K, Jensen R, Katsarava Z, Lipton R, Scher A, Steiner T, Zwart JA (2007). The global burden of headache: a documentation of headache prevalence and disability worldwide. Cephalalgia.

[11] Karikari TK, Charway-Felli A, Hoglund K, Blennow K, Zetterberg H (2018). Commentary: global, regional, and national burden of neurological disorders during 1990–2015: a systematic analysis for the Global Burden of Disease Study 2015. Front Neurol.

[12] Patel S, Potter R, Matharu M, Carnes D, Taylor S, Nichols V, Pincus T, Underwood M, Sandhu H (2019). Development of an education and self-management intervention for chronic headache – CHESS trial (Chronic Headache Education and Self-management Study). J Headache Pain.

[13] Oakley A, Strange V, Bonell C, Allen E, Stephenson J, Team RS (2006). Process evaluation in randomised controlled trials of complex interventions. BMJ.

[14] Bryman A (2006). Integrating quantitative and qualitative research: how is it done?. Qual Res.

[15] Pope C, Mays N (1995). Reaching the parts other methods cannot reach: an introduction to qualitative methods in health and health services research. BMJ.

[16] Ritchie J, Spencer L (2002). Qualitative data analysis for applied policy research. Qual Res Companion.

