# Supplementary material for: The CHESS trial: protocol for the process evaluation of a randomised trial of an education and self-management intervention for people with chronic headache
Source: Trials. 2019 Jun 4;20:323. doi: 10.1186/s13063-019-3372-x (PMC6549347; doi:10.1186/s13063-019-3372-x)
Supplement: Supplementary file 1 — Process evaluation protocol for the CHESS trial: supplementary material. (DOCX 19 kb) [file 13063_2019_3372_MOESM1_ESM.docx]

**Process evaluation protocol for the Chronic Headache Education and Self-Management Study (CHESS) randomised controlled trial: Supplementary material**

**Additional file 2** **Day 1 / Session 4 / Title: Acceptance of chronic headaches – 45mins**

**Aim**: To introduce the concept of acceptance and need for self-management

**CHESS course code: _____ Reviewer: _________________________ Review date: ____________**

**Adherence**

| No. | Item | Adherence | Comments |
| --- | --- | --- | --- |
| 4.1 | Did the facilitator(s) suggest that headaches cause physical sensations and pain but it can be the struggle to manage this pain that causes the overall distress and ask the question, What are their thoughts on this? | Yes (2) Partially (1) No (0) |  |
| 4.2 | Did the facilitator(s) describe or read Lisa’s Case study? | Yes (2) Partially (1) No (0) |  |
| 4.3 | Did the facilitator(s) ask the group to discuss the way she handles the issue? | Yes (2) Partially (1) No (0) |  |
| 4.4 | Did the facilitator(s) ask the group to relate this to their headaches as an uninvited and unwanted guest and how to deal with their pain? | Yes (2) Partially (1) No (0) |  |
| 4.5 | Did the facilitator(s) discuss with the group the idea that Lisa could not control her guest do she would have to learn to get on with him in one way or another? | Yes (2) Partially (1) No (0) |  |
|  | Total competence score --/10 |  |  |
|  | Percentage competence score (Total competence score--/10 x100) |  |  |
| **Instructions:**  When at all possible please rate as ‘Yes’ or ‘No’ If ‘partially’ then write reason in comments box  Questions need not be verbatim (unless specified) as long as content of session is covered. | | | |

**Competence**

| **CHESS** | Item | Competence measure | Comments |
| --- | --- | --- | --- |
| Introduction | Did the facilitator(s) ‘set the scene for the session*? e.g. did they introduce the title, aims or content of the session?* | Evident (2)  Partially evident (1)  Not evident (0) |  |
| Discussion | Did the facilitator(s) encourage individual and group participation? *e.g. did they; encourage individuals to participate, ask open questions, give enough time for the group to answer (rather than answer their own questions)* | Evident (2)  Partially evident (1)  Not evident (0) |  |
| Group climate | Did the facilitator(s) foster a positive group climate? *e.g. Did they say positive things about people ‘that’s a helpful comment’ ’thank you for sharing that’. Did they encourage sharing and exploration of ideas in a non-judgemental way?* | Evident (2)  Partially evident (1)  Not evident (0) |  |
| Summary | Did the facilitator(s) consolidate/embed the group’s learning at the end of the session? | Evident (2)  Partially evident (1)  Not evident (0) |  |
| Linking | Did the facilitator(s) link any of the session to other sessions? | Evident (2)  Partially evident (1)  Not evident (0) |  |
|  | Total competence score Max 10 |  |  |
|  | Percentage competence score (Total competence score/- *100) |  |  |
